# Prevalence of Back Pain in Secondary School Students in an Urban Population: Cross-sectional Study

**DOI:** 10.7759/cureus.2983

**Published:** 2018-07-14

**Authors:** Swati Paranjape, Vaishali Ingole

**Affiliations:** 1 Physiotherapy Department, King Edward Memorial Hospital/Seth Gordhandas Sunderdas Medical College, Mumbai, IND; 2 Physiotherapy Department, Lokamanya Tilak Municipal Medical College, Mumbai, IND

**Keywords:** adolescents, low back pain, prevalence, school students, heavy satchel

## Abstract

Introduction

The prevalence of low back pain (LBP) among secondary school students is increasing. The magnitude of the problem is not well quantified. Evidence shows LBP in adolescents can be a significant risk factor for back pain in adulthood. The present study aimed to determine the lifetime prevalence of LBP among secondary school students from schools of an urban metropolitan city and the prevalence of LBP in the presence of associated factors.

Methods

This cross-sectional analytical study was done using a validated semi-structured questionnaire (N = 555; response rate, 85.3%). Secondary school students between the age of 12 and 15 years from three randomly selected urban secondary schools of Mumbai, India were recruited for the study.

Results

We analyzed the data for prevalence and odds ratios (OR), and we conducted a univariate analysis to determine the significance of LBP prevalence. We found the lifetime prevalence of LBP was high (32.9%). The prevalence of LBP was highly significant (p < .0001) among girls (confidence interval [CI]: 1.5 to 3.2; OR: 2.2), those who felt their school satchel was heavy (CI: 1.7 to 3.5; OR: 2.4) and those who watched television (TV; CI: 0.03 to 0.28; OR: 0.09).

Conclusion

We noted a high prevalence of LBP among adolescents. LBP had a significantly high prevalence among girls and in the presence of factors like a heavy school satchel and watching TV. LBP in adolescence is a significant risk factor in developing back pain in adulthood, and our findings highlight the need for awareness of LBP among stakeholders like educationists, policymakers, medical professionals and parents given the possible detrimental effects on adolescent children.

## Introduction

Low back pain (LBP) is a common health problem in the adult population. However, back pain is seen not only in adults but also in adolescents [1]. This was considered insignificant until two decades ago [2]. Epidemiological research showed that nonspecific LBP is much more frequent among school children than was originally thought [3]. In Brazilian fifth to eighth graders, the period prevalence of LBP over three months was 55.7% [4] while among Nigerian children, the lifetime prevalence was as high as 58% [5]. Among Italian teenagers, the period prevalence of LBP was 20.5% [6], whereas in Belgian students it was 36% [1]. Limited research is available in the Indian population—only two studies from the rural population or small cities were found. Aundhakar et al. [7] reported a 12-month prevalence among Indian rural student population was 57.1%, whereas Kumar et al. [8] reported a one-month prevalence as 22.7%. No studies were found to have assessed an Indian urban metropolitan population.

These studies show that LBP is a significant problem in adolescents and that the magnitude of the problem has increased in recent years.

The risk of developing LBP in adolescents is multi-factorial and includes genetic, behavioral, ergonomic and psychological factors [9]. LBP in schoolchildren can predict LBP in later years [3,10]. The limited research available demonstrates the need to study the magnitude of the problem as well as associated factors to implement reforms and preventive treatment strategies given the possible detrimental effects in this population.

The objectives of this study were to determine the lifetime prevalence of LBP among urban metropolitan secondary school students, identify the factors associated with LBP, and the prevalence of LBP in the presence of these factors.

## Materials and methods

The population under study consisted of secondary schoolchildren aged 12 to 15 years from three randomly selected schools from an Indian metropolitan urban city. In this cross-sectional analytical study, a validated self-reported semi-structured 28-item low back pain questionnaire for schoolchildren was used. The sample size to be used was 550 participants based on the previous studies of lifetime prevalence [11], according to the following formula:

N= \frac{Z^{2}\times p(1-p)}{d^{2}}\\

N = Sample size

Z = Z stats for level of confidence = 1.96

p = expected prevalence with respect to previous studies

d = precision = 10%

The sampling technique was stratified random sampling. Students with congenital or acquired musculoskeletal or neurological conditions or with recent trauma were excluded. Approval was obtained from the institutional ethics committee of Lokmanya Tilak Municipal Medical College. Written informed consent was obtained from the participants’ parents or legal guardians.

Of 650 students, 555 participated in the study for a response rate of 85.3%. The researcher explained the study to the participants, parents, and principals of the schools. The participants completed the questionnaire without discussing it with fellow participants in the presence of the researcher in class during school hours. For items requiring the use of the Visual Analogue Scale (VAS), students were given instructions on the use of the VAS. The questionnaire covered aspects of the students’ self-reported pain, the heaviness of their satchel, time spent watching TV/at the computer/playing video games, use of corrective lenses, history of either parent having LBP, mode of transport to school, as well as their happiness, tiredness, and amount of sleep.

## Results

Data were analyzed using GraphPad Prism for Windows (GraphPad Software, La Jolla, CA, USA). A univariate analysis was performed to find the statistical significance of the prevalence of LBP and its prevalence in the presence of associated factors. Odds ratios (OR) were calculated using the standard formula in the software. The total participants were 555 students: 302 boys (54.4%) and 253 girls (45.5%; mean age, 13 years). A total of 183 students reported having LBP at least once in their lifetime. The lifetime prevalence of LBP was high (32.9%). The prevalence of LBP was significant in the presence of factors such as watching television (TV, confidence interval [CI]: 0.03 to 0.28; OR: 0.09), a heavy school satchel (CI: 1.7 to 3.5; OR: 2.4) and female gender (CI: 1.5 to 3.2; OR: 2.2). A total of 532 students were watching TV every day (95.8%). Among these, 164 students complained of LBP (30.8%) while 368 students had no LBP (69.1%). Regarding satchel weight, 257 students (46.3%) felt their school satchel was heavy. Of these, 112 (43.5%) complained of LBP while 145 (56.4%) did not have LBP. Among the 253 girls, 108 (42.6%) reported LBP whereas, among the 302 boys, only 75 (24.8%) reported LBP. Among the 183 students who reported LBP, 63.3% had a history of at least one of their parents having LBP. A total of 301 students (72.3%) reported at least one of their parents having LBP. Of these students, 116 (38.5%) students reported LBP. Table 1 summarizes these findings.

**Table 1:** Factors associated with LBP and their prevalence. * - Statistically significant values determined via chi-square test (P < .0001). The row or column association is statistically significant. # - Yates correction (P < .0001). LBP: Low back pain; TV: Television.

Factors associated with LBP	Students having LBP	Students not having LBP	Prevalence (%)	95% confidence interval	Odds ratio
Spectacles					
Yes	41	96	29.92	0.54 to 1.26	0.83
No	142	276			
Watching TV					
Yes	164	368	*30.83#	0.03 to 0.28	0.09
No	19	4			
Playing video games/computers					
Yes	142	301	32.05	0.52 to 1.26	0.81
No	41	71			
Heavy satchel					
Yes	112	145	*43.57	1.71 to 3.55	2.47
No	71	227			
At least one parent having LBP					
Yes	116	185	38.54	1.21 to 2.51	1.75
No	67	187			
Girls	108	145	*42.69	1.57 to 3.23	2.25
Boys	75	227			

A total of 443 students (79.8%) played computer or video games every day. Of these, 142 (32%) had LBP while 301 (67.9%) had no LBP. A total of 137 students wore spectacles. Of these, 41 students (29.9%) had LBP whereas 96 students (70%) had no LBP. Out of 183 students reporting LBP, 108 students were unhappy (59%). Table 2 and Table 3 summarize these findings.

**Table 2:** Self-reported responses to the questionnaire by participants. LBP: Low back pain; TV: Television.

	N	Low back pain (N)	Low back pain (%)	No low back pain (N)	No low back pain (%)
Boys	302	75	24.83	227	75.16
Girls	253	108	42.68	145	57.31
Spectacles	137	41	29.92	96	70.07
Watch TV	532	164	30.82	368	69.17
Play video games	443	142	32.05	301	67.94
Parents having LBP	302	116	38.41	186	61.58
Heavy satchel	257	112	43.57	145	56.42
Walk to school	416	142	34.13	271	65.14

**Table 3:** Percentage distribution of factors associated with LBP (N = 555). LBP: Low back pain; TV: Television.

Factor	Low back pain (n)	Low back pain (%)
Boy	75	40.98
Girl	108	59.01
Sought medical help	38	20.76
At least one of the parent has LBP	116	63.38
Unhappy	108	59.01
Wears spectacles	41	22.40
Watches TV for more than 1 hour/day	164	89.61
Plays video game/computer for more than 1 hour/day	142	77.59
Heavy satchel	112	61.20

## Discussion

From this cross-sectional study, we concluded the prevalence of LBP is very high in urban secondary school students. The prevalence of LBP is significantly higher in girls than boys, among those who watch TV regularly, and those who feel their school satchel is heavy.

The lifetime prevalence of LBP is high. At a prevalence of 32.9%, our findings align with those of the meta-analysis by Calvo-Muñoz et al. [11] that reported a 39.9% lifetime prevalence. Various other studies have also reported a high prevalence of LBP in the adolescent population [1,3-6]. However, a large variation in prevalence rates was seen in these studies. Calvo-Muñoz et al. [11] attributed this variation to differences in the age group of the samples, the sample sizes, the definitions of LBP, the LBP recall period, data collection strategies and the methodologies.

Our secondary outcome measure was factors associated with LBP. The first factor significantly associated with a high prevalence of LBP was watching TV regularly. Noll et al. [4] reported the probability of developing back pain doubles among those children who watched TV for two hours a day. Watching TV is mostly done while sitting or even lying on a couch. Prolonged sitting can cause disc compression and discomfort [4]. The amount of time spent watching TV, prolonged sitting, bad posture, or lack of physical activity may be associated with LBP related to watching TV [2]. The present study endorses these findings as there were only 228 children that played sports while almost all watched TV regularly, indicating lack of physical activity and a sedentary lifestyle.

The second factor found to have a significant prevalence in LBP was a heavy satchel. Previous studies have also established the occurrence of LBP among children who feel their school satchel is heavy [4,12]. Most international standards accept a satchel weighing 10% to 15% of the body weight of the child [12,13]. Carrying higher weights than the recommended standards can cause serious health problems in these children [14], bring about abnormal changes in gait performance [15], and cause modifications to natural standing postures [16,17]. In previous studies where an objective assessment of the weight of the schoolchildren and their backpacks was performed, the prevalence of LBP was 42% [12]. Both walking to school with backpacks and using heavy backpacks had a high prevalence and increased the relative risk of developing back pain [10]. The findings in our present study are in agreement with this research as most of our study subjects also carried their satchel on their backs, and 34.1% of our study participants who walked to school reported LBP.

The third significant factor was gender, specifically being a girl. In the present study, LBP among female students (42.6%) was high compared to the incidence of LBP among boys (24.8%). Many researchers have identified this as a factor [12,18] and have attributed the difference to the hormonal changes experienced during puberty affecting attitudes or perception of pain [6]. Balagu et al. [2] identified female gender as a significant risk factor for LBP.

In our study, one additional factor—a parental history of LBP—had a high prevalence of association with LBP among participants. Though seemingly less researched, a review of the literature indicates children of a responsible guardian or parent that has back pain have a higher likelihood of developing back pain themselves [1,2,4]. Researchers have attributed this to genetic, behavioral, and psychological factors [4]. As the present study gathered information from the students themselves and not parents, it may have missed or biased the data on this factor so we cannot endorse this finding.

This study also has certain limitations. First, since the study population was urban secondary school students, the findings may not be applicable in a rural population. Next, the study was cross-sectional, so the causal association of these factors with LBP cannot be established. The limitations of the study suggest future research into the causal association of these factors with LBP assessed through longitudinal cohorts. Finally, gender-specific differences can also be studied further by psychometric and hormonal analysis.

## Conclusions

Our study provides alarming evidence of the high prevalence of LBP in an urban adolescent population. Given the association of LBP during adolescence with developing back pain as an adult, LBP in adolescents warrants the attention of all stakeholders including educators, policymakers, medical professionals, and parents. This also highlights the need for preventive care programs, encouraging an active lifestyle, and ergonomic modifications specific to adolescents.
